# Do efforts to standardize, assess and improve the quality of health service provision to adolescents by government-run health services in low and middle income countries, lead to improvements in service-quality and service-utilization by adolescents?

**DOI:** 10.1186/s12978-015-0111-y

**Published:** 2016-02-06

**Authors:** Venkatraman Chandra-Mouli, Subidita Chatterjee, Krishna Bose

**Affiliations:** 1 Department of Reproductive Health and Research, World Health Organization, Avenue Appia, Geneva, 1211 Switzerland; 2 Independent expert, Kolkata, India; 3 Department of Maternal Newborn Child and Adolescent Health, World Health Organization, Geneva, Switzerland

**Keywords:** Quality of care in adolescents, Adolescent friendly health services, Quality standards and criteria, Quality assessment, Quality improvement

## Abstract

**Background:**

Researchers and implementers working in adolescent health, and adolescents themselves question whether government-run health services in conservative and resource-constrained settings can be made adolescent friendly. This paper aims to find out what selected low and middle income country (LMIC) governments have set out to do to improve the quality of health service provision to adolescents; whether their efforts led to measurable improvements in quality and to increased health service-utilization by adolescents.

**Methods:**

We gathered normative guidance and reports from eight LMICs in Asia, Africa, Central and Eastern Europe and the Western Pacific. We analysed national quality standards for adolescent friendly health services, findings from the assessments of the quality of health service provision, and findings on the utilization of health services.

**Results:**

Governments of LMICs have set out to improve the accessibility, acceptability, equity, appropriateness and effectiveness of health service provision to adolescents by defining standards and actions to achieve them. Their actions have led to measurable improvements in quality and to increases in health service utilisation by adolescents.

**Conclusions:**

With support, government-run health facilities in LMICs can improve the quality of health services and their utilization by adolescents.

## Background

Government-led efforts are underway in a number of low and middle income countries (LMIC) to make health service provision adolescent friendly. This paper examines whether in fact these efforts make health services adolescent friendly and result in increased use of health services by adolescents.

In 2001, based on a review of the available evidence and on the experiences of organizations from around the world, WHO called for countries to undertake efforts to make health services adolescent friendly: ‘All adolescents should be able to access promotive, preventive and curative health services relevant to their stage of maturation and life circumstances’ (consensus statement 2) [1]. WHO’s call noted that ‘for a variety of reasons, adolescents in many places are unable to obtain the health services they need’ (consensus statement 3) and outlined promising approaches that could be used to overcome these barriers and increase health service utilization by adolescents (consensus statement 6).

In 2007, WHO’s recommendation was reiterated in a paper published in The Lancet. Based on an updated review of the literature the authors concluded: ‘Enough is known that a priority for the future is to ensure that each country, state and locality has a policy and support to encourage provision of innovative and well-assessed youth-friendly health services’ [2].

In international conferences and in other fora, government-run clinics and hospitals are described as being unwelcoming to adolescents, and health workers in government-run health facilities are perceived to be judgemental and unfriendly, and to lack the clinical and interpersonal competencies needed to provide health services to adolescents effectively and with sensitivity [3]. And researchers and implementers question whether government-run health facilities and health workers can be made adolescent friendly at all [4].

To respond to these questions and concerns, we set out to answer the following three questions:

1. What did LMIC governments set out to do, to improve the quality – including the friendliness – of health service provision to adolescents? (Here and henceforth in this paper, quality includes friendliness.)
2. Did their efforts lead to improvements of quality of health service provision to adolescents?
3. Did these improvements in quality lead to increased health service-utilization by adolescents?

## Methods

Beginning in 2001, WHO worked with partners within and outside the United Nations system to support Ministries of Health in LMIC standardize, assess and improve the quality/expand the coverage of health service provision to adolescents in primary-level government-run health facilities in nearly 25 countries in Asia, Africa, Central and Eastern Europe and the Western Pacific (Table 1).

**Table 1:** WHO-supported efforts in LMIC to standardize, assess and improve the quality of health service provision to adolescents in primary-level government-run health facilities

The problem:
In many low and middle income countries, there was widespread recognition that adolescents were not obtaining health services, and that this led to missed opportunities to prevent health problems and respond to them when they occurred.
Nongovernment organizations (NGOs) were the first to respond to this need by establishing *stand-alone* Adolescent Friendly Health Services (AFHS). There was no agreed upon definition of what AFHS meant, although many NGO efforts aimed to make health workers non-judgemental and empathic, make health facilities welcoming and to ensure confidentiality.
Ministries of Health called for WHO guidance to draw upon experiences gained in small-scale and often time-limited nongovernmental projects to make government-run health workers and health facilities more responsive to adolescents.
WHO’s response to the problem:
Gathering and synthesizing evidence:
1. WHO defined attributes of Adolescent Friendly Health Services based on implementation experience and research evidence and placed these attributes in a quality of care framework [5]:
• Accessible: Adolescents *are able to* obtain the health services that are available
• Acceptable: Adolescents *are willing to* obtain the health services that are available
• Equitable: *All adolescents, not just some groups of adolescents*, are able to obtain the health services that are available
• Appropriate: The *right health services (i.e. the ones they need)* are provided to them
• Effective: The *right health services are provided in the right way*, and make a positive contribution to their health
*2.* WHO developed and tested tools to standardize, assess and improve the quality and expand the coverage of health service provision to adolescents in LMIC [5–7].
Taking evidence to action:
Beginning in 2001, WHO worked with partners within and outside the United Nations system to support Ministries of Health to:
1. Develop national quality standards using the five-step process outlined in WHO’s tool *Making health services adolescent friendly: developing national quality standards for adolescent friendly health services* [5]*;*
2. Improve the quality of health service provision through complementary actions at the national, district/municipal and local levels *as described in annex 2 of the above tool* [5]*;*
3. Assess the quality of health service provision using national adaptations of WHO’s toolkit: *Quality assessment guidebook: A guide to assessing health services for adolescent clients* [6]*;*
4. Assess the coverage of health services using national adaptations of WHO’s tool kit: *Coverage assessment guidebook: A guide assessing the coverage of quality health services for adolescents* [7]*;*
5. Share the findings of the quality and coverage assessments at the national level, and use them to address areas of weakness as part of the ongoing effort to improve the quality of health service provision to adolescents.

We reviewed published and unpublished documents - normative guidance and reports emanating from these LMIC and selected eight in which we had information to answer the questions we set out to answer - Bangladesh, India, Indonesia, Malawi, Moldova, Mongolia, Tanzania and Ukraine.

To answer the first question, we analysed national quality standards and accompanying criteria for adolescent friendly health services developed by each of these countries against WHO’s dimensions of quality adolescent friendly health services. Depending on whether the quality dimensions had been named in the standard statements, and depending on how adequately they were addressed in the accompanying criteria, we placed the countries in four categories – highly adequate, moderately adequate, not-fully adequate and absent. To answer the second question, we analysed findings from the assessments of the quality of health service provision carried out under the auspices of the Ministries of Health of these countries. Depending on compliance with the required standard of quality, we placed them in three categories: ≥ 70 % compliance = performing well; 40– 69 % = need some improvement and ≤39 % = need considerable improvement. To answer the third question, we analysed findings on the uptake of health services by adolescents (10–19 years) and youth (20–24 years) from health facility-based service statistics and from community-based coverage studies.

## Results

1. What did LMIC governments set out to do, to improve the quality of health service provision to adolescents?
  Our analysis of national standards and criteria showed that governments of the eight countries set out to improve the accessibility, acceptability and effectiveness dimensions of quality with reasonable adequacy. However, they addressed the appropriateness and equity dimensions less adequately. Selected details are provided below:
  The **accessibility** and **acceptability** dimensions of quality were highly adequately addressed in five of the eight countries and moderately adequately addressed in another three of them.
  The **equity** dimension of quality was highly addressed in one of the eight countries, moderately adequately addressed in one, not fully adequately addressed in three and absent in three.
  The **appropriateness** dimension of quality was highly adequately addressed in two of the eight countries, moderately adequately addressed in two, not fully adequately addressed in three and absent in one.
  The **effectiveness** dimension of quality was highly adequately addressed in five of the eight countries, moderately adequately addressed in two and not fully adequately addressed in one.
    Table 2 contains a detailed analysis of this.

**Table 2:** Analysis of how adequately the WHO dimensions of quality are addressed in the national standards for quality health service provision for adolescents of selected countries

Country	Accessibility	Acceptability	Equity	Appropriateness	Effectiveness
Bangladesh [10]	Highly adequate	Highly adequate	Highly adequate	Moderately adequately	Moderately adequate
	Standards 1, 2,	Standards 3, 4, 6	Standard 5	Standard 8	Standard 9
India [11]	Highly adequate	Moderately adequate	Not fully adequate	Highly adequate	Moderately adequate
	Standards 1, 5, 6	Standard 3	Standard 1	Standards 1, 2	Standard 2
Indonesia [12]	Moderately adequate	Highly adequate	Absent	Not fully adequate	Highly adequate
	Standards 3, 4	Standards 1, 2, 3, 4		Standard 2 ,	Standards 1, 2, 5
Malawi [13]	Moderately adequate	Moderately adequate	Absent	Not fully adequate	Highly adequate
	Standards 2, 3	Standard 3, 4		Standard 2	Standards 1, 4, 5
Moldova [14]	Highly adequate	Moderately adequate	Moderately adequate	Absent	Not fully adequate
	Standards 1, 2, 4	Standard 3	Standard 6		Standard 5
Mongolia [15]	Highly adequate	Highly adequate	Absent	Highly adequate	Highly adequate
	Standards 3, 4	Standards 2, 3, 4		Standards 1, 4	Standards 1, 3
Tanzania [16]	Highly adequate	Highly adequate	Not fully adequate	Not fully adequate	Highly adequate
	Standards 1, 7	Standards 3, 6	Standard 2	Standard 6	Standards 2, 3, 4, 5,
Ukraine [17]	Moderately adequate	Highly adequate	Not fully adequate	Moderately adequate	Highly adequate
	Standards 3, 5, 9	Standards 3, 4, 9	Standard 5	Standards 7, 8	Standards 2, 6, 10
2. Did the efforts of LMIC governments lead to improvements in the quality of health service provision to adolescents?
  Our analysis of the assessments of the quality of health service provision in the eight countries showed that while the assessments were carried out in different contexts and by different organizations, they all used the same combination of approaches - interviews with health facility managers, health service providers, support staff and adolescent patients/clients, and observation of health service delivery points. Further, while there were variations in the levels of improvement of the different dimensions of quality in the eight countries, efforts to improve quality of health service provision, led to observable and measurable improvements. Selected details are provided below:
**Accessibility**: Accessibility was assessed in all eight countries. Various criteria of this dimension were rated as performing well in five countries, needing some improvement, in five countries and needing considerable improvement in four countries.
**Acceptability**: Acceptability was also assessed in all eight countries. The criterion ‘health facilities have a welcoming and friendly ambience’ was rated as performing well by seven countries; Moldova rated it as needing some improvement. Six countries assessed privacy and five assessed confidentiality. On privacy, three of the six performed well, two of the six needed some improvement, and one needed considerable improvement. On confidentiality, one of the five performed well, while the other four needed some improvement.
**Appropriateness**: Only four countries measured appropriateness. On the provision of a specified package of services, one was rated as performing well, and two as needing some improvement. For the fourth country (Indonesia), some interventions within the specified service package were assessed as performing well or needing some improvement while the functioning of the referral system was rated as requiring considerable improvement.
**Equity**: The equitable provision of health services was assessed in only two countries. It was rated as needing some improvement in both.
**Effectiveness**: The various criteria of this dimension were also assessed in all eight countries. Regarding the training of health service providers, five of the eight countries were assessed with one of them rated as needing some improvement and the other four as needing considerable improvement. On data management and use, only three countries were assessed with one of them rated as needing some improvement and the other two as needing considerable improvement.
  Table 3 contains an analysis of the acceptability dimension of quality in all eight countries. (Details of the assessment of other dimensions are available on request).

**Table 3:** Analysis of the context in which the quality of health service provision to adolescents was assessed, who assessed it, what the objectives of the assessment were, how the assessment was done, and findings of the assessment

Country	What was the context in which quality was assessed?	Who did the quality assessment?	What were the objectives of the quality assessment?	What were the findings of the quality assessment on the acceptability dimension of quality (i.e. adolescents are willing to obtain the health services that are available)?
			What methods were used in the quality assessment?	
Bangladesh [18]	National quality assessment study	National research institution with support from WHO	Objectives: To assess compliance of the quality of health service provision with the 10 national standards and to determine if there were differences between intervention and comparison health facilities.	Performing well: Young People feel comfortable with the surroundings and procedures of Health Service Delivery Points 74% versus 66%.
				Need some improvement: The privacy and confidentiality of all young people who visit delivery points is maintained 63% versus 58%
				Service providers are motivated to provide health services to young people in a youth friendly manner 66% versus 63%.
			Methods: Quality assessment in 44 intervention and 44 comparison facilities	
India (Haryana state) [19]	State level quality and coverage assessment study	Nongovernment organization with support from WHO	Objectives:To assess compliance with national standards and to determine if there were differences between intervention and comparison health facilities.	Performing well: Adolescents find the environment at health facilities conducive to seek services 86% versus 33%
				Service providers are sensitive to the needs of adolescents and are motivated to work with them 94% versus 59%
			Methods: Quality assessment in 10 intervention and 10 comparison health facilities	
			Both samples consisted of 2 Primary Health Care centers and 8 Sub-centers.	
Indonesia [20]	National level quality assessment survey	Ministry of Health with support from WHO	Objectives:To assess compliance with national quality standards.	Performing well: Adolescents are satisfied with the services 73%, health providers have positive attitudes about working with adolescents 73%
			Methods:Cross sectional study of 62 adolescent friendly Primary Health Centers	Need some improvement: Adolescent perceive that their confidentiality will be respected 42%
				Need considerable improvement: Adolescents feel comfortable about using the health services 38%; staff ae oriented on adolescent friendly health services 27%; services are provided outside regular hours 9%; adolescents are engaged in planning 10%; adolescents are engaged in monitoring 3%; mechanisms are in place to ensure privacy 18%
Malawi [21]	National level quality assessment study to assess readiness for accreditation	National youth council of Malawi and Ministry of Health, Malawi with the support of UNFPA	Objectives:To assess compliance with national quality standards.	Performing well: Privacy and respect for adolescents 83%
			Methods:Cross-sectional study involving 266 randomly sampled sites	Need some improvement: Involvement of young people 60%; availability of educational materials 50%; support staff oriented on youth friendly health services 51%
				Need considerable improvement: Recreational materials are available 37%
Moldova [22]	National level quality assessment study	National working group with support from WHO	Objective: To study the compliance of Youth Friendly Health Services with national quality standards.	Need some improvement: Service providers respect youth confidentiality and privacy 68%
			Methods: Assessment of 12 Youth Friendly Clinics	
Mongolia [23]	National level quality assessment study	Consultant team engaged by Ministry of Health and supported by WHO	Objectives: To compare the quality of health facilities in intervention and comparison sites.	Performing well: Written confidentiality policy 86.3% versus 7.1%; providers respect adolescent opinions 82.1% versus 47.6% ; Information Education and Communication services provided 98% versus 75%,; receptionists are friendly 92.9% versus 82.9%, doctors are friendly 96.1% versus 86.5%; waiting area is comfortable and convenient 71.7% versus 18.8%
			Methods: Assessment of quality in 82 sites (51 intervention sites and 31 comparison sites), to determine which dimensions of quality and most important for client satisfaction.	
				Need some improvement: Toilets are of good quality 53.2% versus 42.9%,youth participation present 45.5% versus 27.1%, confidentiality policy is posted 48% versus 0%; clients are satisfied with services 45% versus 33%
				Need considerable improvement: Long waiting time (15.4% versus 10.2%), written policy on patient consent 13.7% versus 0%
Tanzania [24]	National level quality assessment	Nongovernment Organization on behalf of the Ministry of Health	Objectives: To assess compliance with national quality standards.	Performing well**:** Service Delivery Points ensure privacy and confidentiality78%; clean and appealing 72%
			Methods: Cross-sectional survey involving 90 health facilities randomly chosen. Nine districts of mainland Tanzania were involved, covering all 8 Ministry of Health and Social Welfare zones.	
Ukraine [25]	National level quality assessment study	Academic institution with the support of the Ministry of Health and UNICEF.	Objectives: To compare progress made in compliance with national standards, with the findings of a previous assessment.	Performing well: Friendly behaviour from registration staff 90%; mentioned similar treatment by physicians and psychologists 95%; clients reported specialists were not distracted by external interruptions 97
	Repeat Evaluation report		Methods: Repeat assessment in 23 Health Centres	

Legend: ≥ 70%= Performing well, 40%- 69% = need some improvement and ≤ 39% need considerable improvement
3. Did the improvements in the quality of health facilities lead to increased health service-utilization by adolescents?
  We gathered and analysed reports from all eight countries. In two countries health facility based service statistics were available at two time periods (Malawi and Mongolia) and 3 time periods (Ukraine). In Mongolia, health facility based service statistics were available from intervention and comparison sites, from a cross-sectional survey. In Bangladesh, India and Tanzania, community-based coverage studies compared self-reported service use in health facilities in intervention and comparison areas. In Moldova, community-based coverage studies were carried out at two time-periods but without comparison facilities. In Indonesia, no utilisation data were available from health facilities or through community-based coverage studies, but a Ministry of Health supported project in Aceh province showed improvements in quality and increased health service utilisation. Health service utilization was lower in intervention health facilities than in comparison health facilities in Bangladesh, through the difference was not statistically significant. In the other seven countries improvements in service quality were associated with increases in service utilization. Table 4 contains an analysis of this.

**Table 4:** Analysis of the context in which the health service utilization by adolescents was measured, who measured it, how the measurement was done, and what the findings of the measurement were

Country	Context in which health service utilization by adolescents was measured	Who did the measurement?	Methods used in the measurement	Results of the measurement of service utilization by adolescents
Bangladesh [18]	National quality and coverage assessment	A research institution with support from WHO	Household coverage survey	Though 49 % of the youth in the intervention areas and 54 % in the comparison areas reported visiting a health service provider in the period six months prior to the survey, this difference was not significant
India [19]	State level quality and coverage assessment study	Nongovernment organization with support from WHO	Household coverage survey carried out in the catchment area of health facilities - 10 village clusters around 10 intervention sub-centres and 10 village clusters around 10 comparison sub-centres	For government-run health facilities, the utilization of health services in the intervention health facilities was higher than that in than the comparison facilities.
Indonesia [20]	Managers and staff of 62 health facilities surveyed reported that service-utilization by adolescents was increasing but there was no data to back up their impressions. (20)			
	Data on health service utilization was not available from the Ministry of Health. Data from a project in Aceh province, Indonesia showed both improvements in quality and increases in service/utilization [26].			
Malawi [21]	District level project on improving access to sexual and reproductive health services for adolescent girls	Joint United Nations programme on adolescent girls in conjunction with the Ministry of Health at the national and Chikwawa district level	Assessment of service statistics	In 2011, there were 12 visits by girls aged 10–14 years and 330 visits by girls aged 15–19 years. In 2012 there were 163 visits by girls aged 10–14 years and 512 visits by girls aged 15–19 years.
Moldova [22]	External review of a project to scale up adolescent friendly health services nationwide	Independent team of consultants engaged by the Swiss Development Cooperation Agency with support from the Ministry of Health	Household coverage survey and assessment of service statistics	In 2009 - 5 % young people reported using youth friendly health services (30 % boys and 70 % girls). In 2012, 12 % (38.7 % boys and 61.3 girls) did so.
Mongolia [23]	Nationwide survey of quality and coverage of health service provision for adolescents	Consultant team engaged by Ministry of Health and supported by WHO	Assessment of service statistics from 82 health facilities complemented by a school based coverage survey	The number of boys aged 14 – 18 who used services increased from 15,575 in 2008) to 30,741 in 2010. The number of girls aged 14 – 18 years who used services increased from 29,852 in 2008 to 57,236 in 2010. Similarly, the number of boys aged 18–24 s who used services increased from 6,914 in 2008 to 17,381 in 2010. And the number of girls aged 18 –24 who used services increased from 13,876 in 2008 to 32,072 in 2010.
Tanzania [24]	Data on health service utilization was not available from the Ministry of Health. Data from a multisite project in the country showed both improvements in quality and increases in service/utilization [27].			
Ukraine [25]	National level quality assessment study - repeat evaluation	Academic institution with the support of the Ministry of Health and UNICEF	Analysis of service statistics	Data emanating from evaluations carried out in 2008, 2009 and 2010, show a steady trend in the increase in service utilization both for the younger and the older age groups of adolescents.

## Discussion

Our analysis of normative guidance documents from eight LMIC from different parts of the world shows that the governments of these countries have set clear expectations for the quality of health service provision to adolescents at the primary care level. Our analysis of the quality of health service provision in these countries showed measurable improvements, although these were uneven. Our analysis of health facility-based service statistics and community-based coverage studies shows that improvements in the quality of health service provision are accompanied by improvements in service utilization by adolescents.

Our results clearly show that the quality of health service provision to adolescents in government-run health facilities and their utilization can be improved in different social, economic and cultural contexts. A limitation of our study is that, while we analysed what governments *set out to do* to improve the quality of health service provision, we were not able to assess what they actually did. Thus, we cannot comment on what actually occurred to bring about these improvements.

Other authors have shown that the quality of health service provision to adolescents by government-run health facilities in LMIC and service utilization by adolescents can be improved. But these improvements have been brought about in the context of operations research or well-funded and tightly-managed projects [8, 9]. Our findings show that such improvements are possible even in the context of routine programming e.g. in India, Tanzania and Ukraine.

A strength of this study is that we assembled data from published reports of quality assessment, and of service utilization and coverage assessment in different contexts by different organizations, including academic institutions, nongovernment organizations and consultant teams. While the attributes assessed and the methods used were broadly similar across assessments, another strength, the level of rigor varied greatly. Given this, a meta/analysis was not possible. While this is an important limitation, it is also reflective of the reality of programmatic monitoring and evaluation activities for AFHS programmes in LMICs.

## Conclusions

With support, governments in LMIC have defined quality standards for health service provision to adolescents and the actions needed to achieve them. Actions to achieve these standards have led to increases in the quality and in the utilization of government-run health services by adolescents in very different contexts. This study provides a sound basis for decision makers in national and international institutions to invest in efforts to expand the reach of good quality health services to world’s adolescents.
